# 

**Published:** 1871-04

**Authors:** 


### CONTENTS.

ORIGINAL COMMUNICATIONS.

PAGE.

Diseases peculiar to Females. By J. P. LOGAN, M. D., Atlanta, Ga..... 1

Inhalations in Pectoral Diseases. By J. G. WESTMORELAND, M. D., Atlanta, Ga........ 6

Nasal Spritz—a New Apparatus for treating diseases of the Nasal Cavity, with Remarks. By WM. ABRAM LOVE, M. D., Atlanta, Ga......... 10

Excision of a Large Cervical Glandular Tumor. By J. WISTOR VANCE, L. R. C. S. & P. E........ 17

Extracts from the Minutes of the Atlanta Academy of Medicine........ 19

SELECTIONS.

Manila Paper, as a Material for Splints and the immovable Apparatus. By R. O. COWLING, M. D., Demonstrator of Anatomy in the University of Louisville........ 22

Two Cases of Luxation of the Elbow Backward. By LEWIS A. SAYRE, M. D., Professor of Orthopedic and Clinical Surgery, Bellevue Hospital Medical College........ 28

Clinical Lectures on Medicine. Delivered at the Medical Hospital. By CHARLES MURCHISON, M. D., LL. D., F. R. S. Lecture 4.......... 32

Combined Solution of Pepsine and Pancreatine........ 38

Preservation of Vaccine Crusts........ 40

Extraordinary Case of Enlarged Spleen........ 41

The Pathology of Cerebral Hemorrhage........ 43

EDITORIAL.

Atlanta Medical and Surgical Journal........ 45

Atlanta Academy of Medicine........ 47

Atlanta Medical College........ 50

Cerebro-Spinal Meningetis........ 51

Manila Paper Splint........ 52

To Subscribers........ 54

MISCELLANEOUS.

The Royal College of Physicians on Small Pox and Vaccination........ 55

Belladonna in arresting Nocturn l Incontinence of Urine........ 56

Digitalis in Delirium Tremens........ 57

Rapid Cure of Buboes........ 58

Treatment of Inflamed Testicle by Puncture........ 58

An Insurance Incident........ 59

On the Nature of the Spleen........ 59

Fracture of Anterior Spinous Process of Ilium by Muscular Contraction. 60

Fatality of Small Pox at Different Ages........ 60

Chloral and Strychnine........ 61

Death of Dr. Keith, of Aberdeen........ 61

Incontinence Cured by Chloral Hydrate........ 61

Strangulated Hernia Reduced by Application of Cold Water........ 62

Subnitrate of Bismuth in Diarrhœa of Children........ 62

Tonsillotomy........ 62

Peculiar Case of Scarlatina........ 62

